# Distributions of economically important nonindigenous forest pests in the conterminous United States

**DOI:** 10.1002/ecy.70551

**Published:** 2026-10-05

**Authors:** Katie A. Grong, Randall S. Morin, Yue S. Li, Ningning N. Kong, Susan J. Crocker, Frank J. Krist, Andrew M. Liebhold, Songlin Fei

**Affiliations:** ^1^ Department of Forestry and Natural Resources Purdue University West Lafayette Indiana USA; ^2^ USDA Forest Service, Northern Research Station York Pennsylvania USA; ^3^ Libraries and School of Information Studies Purdue University West Lafayette Indiana USA; ^4^ USDA Forest Service, Northern Research Station St. Paul Minnesota USA; ^5^ USDA Forest Service, Forest Health Protection Fort Collins Colorado USA; ^6^ USDA Forest Service, Northern Research Station Morgantown West Virginia USA; ^7^ Faculty of Forestry and Wood Sciences Czech University of Life Sciences Prague Prague Czech Republic

**Keywords:** damage, forest insect and disease, mortality, pathway, pest invasion, spread

## Abstract

The geographical distributions of established nonindigenous forest insects and pathogens have historically not been well documented nor centrally aggregated, the absence of which has contributed to constrained efforts to manage and minimize resultant damages. To address this need, we present the construction of a centralized database accessible via a publicly available web portal, the Alien Forest Pest Explorer. All nonindigenous forest insect and pathogen species known to cause economic impacts in the conterminous U.S. were selected for inclusion in the database. The occurrence of these established pests, which is derived from a variety of data streams including reports by state and Federal agencies, the scientific literature, and biodiversity databases, is recorded at the county level. In addition to data on geographical distribution of the pests, the database also provides county‐level data that quantifies the volume of host trees species derived from forest inventory data. Overall, the data and analyses presented here provide baseline information regarding the distribution of nonindigenous forest pests and changes in host resources. Our database can also help to provide a more complete understanding of the impacts of nonindigenous forest pests on U.S. forests and can be used as a public resource in supporting regional invasive forest pest research and management. The data are available for reuse under CC0 1.0 Universal licensing; however, we appreciate the citation of this data paper when reusing the dataset.

## CONFLICT OF INTEREST STATEMENT

The authors declare no conflicts of interest.

## Supporting information


Data S1.

## Data Availability

Data are available in Data S1. These data are also available in the Purdue University Research Repository at https://doi.org/10.4231/HWQF-V087.

