# Supplementary material for: Distributions of economically important nonindigenous forest pests in the conterminous United States
Source: Ecology. 2026 Oct 5;107(10):e70551. doi: 10.1002/ecy.70551 (PMC13636186; doi:10.1002/ecy.70551)
Supplement: Supplementary file 1 [file ECY-107-e70551-s001.zip › Metadata_S1.pdf]

Data Paper published in *Ecology*, an Ecological Society of America journal

Metadata S1

Distributions of economically important non-indigenous forest pests in the conterminous United States

Katie A. Grong, Randall S. Morin, Yue S. Li, Ningning N. Kong, Susan J. Crocker, Frank J. Krist, Andrew M. Liebhold, and Songlin Fei

Introduction

The impacts of non-indigenous pests on forest ecosystems have been elevating with increasing numbers of established non-indigenous species and their expanding geographical distributions. The accumulation of these established species is due in large part to increases in global trade and travel which provide pathways by which individuals are inadvertently introduced to new areas (Liebhold et al. 2017, Brockerhoff and Liebhold 2017). In North America, most non-indigenous pest species continue to expand their ranges (Liebhold et al. 2013, Hudgins et al. 2017); therefore, accessible information on the presence of these damaging pests is an essential part of understanding and managing the spread and impact on forest ecosystems.

There are over 400 non-indigenous forest pests established in the U.S, most of which have no record of any significant impact on forest ecosystem dynamics. However, there is a subset of these pests that cause noticeable damage, often expressed through significant tree mortality, canopy thinning, and decreased growth and regeneration (Aukema et al., 2010, Lovett et al., 2016). In turn, these damages can have severe implications for both carbon sequestration capacity and biomass loss (Fei *et al*. 2019) and can threaten tree species that are ecologically, economically, or culturally important (Trotter et al. 2013, Morin and Liebhold 2016, Siegert et al. 2023). Given the significant impacts of these species on forest ecosystem services (Fei et al. 2019), the availability of information on both pest and host distributions throughout the conterminous U.S. can improve timeliness and effectiveness of regional management.

The compiled pest distribution dataset presented here can be used for various purposes such as management decision making, policy making, public education, and scientific research. Pest distribution data have been analyzed in several scientific papers, such as Liebhold et al. (2013), Guo et al. (2019), and Ward et al. (2022). We built an interactive data dashboard, AFPE, which

allows users to view the distribution of the pests, statistics about host tree densities, and dynamics relative to the spatial distribution of pests and to filter the results spatially on the map. For examples, the dashboard provides an overview of all pests detected in each county (Figure 1). After clicking on the counties on the map in the AFPE dashboard, the names of the species detected (field "AllPest") are shown in a pop-up window. When the corresponding pest is selected from the dropdown menu, the presence of the pest species (field "DCA[xxxxx]") is mapped (see example in Figure 2). In addition, users can also quickly examine the trend in host volume and mortality across different areas of the pest or host's ranges by zooming in to areas of interest and panning the map. The data will then be auto updated based on the extent of the map. Moreover, users can filter by selecting groups of counties with the selection tool built in the dashboard. Finally, users can filter, share, and embed customized maps for public education and management decision making.

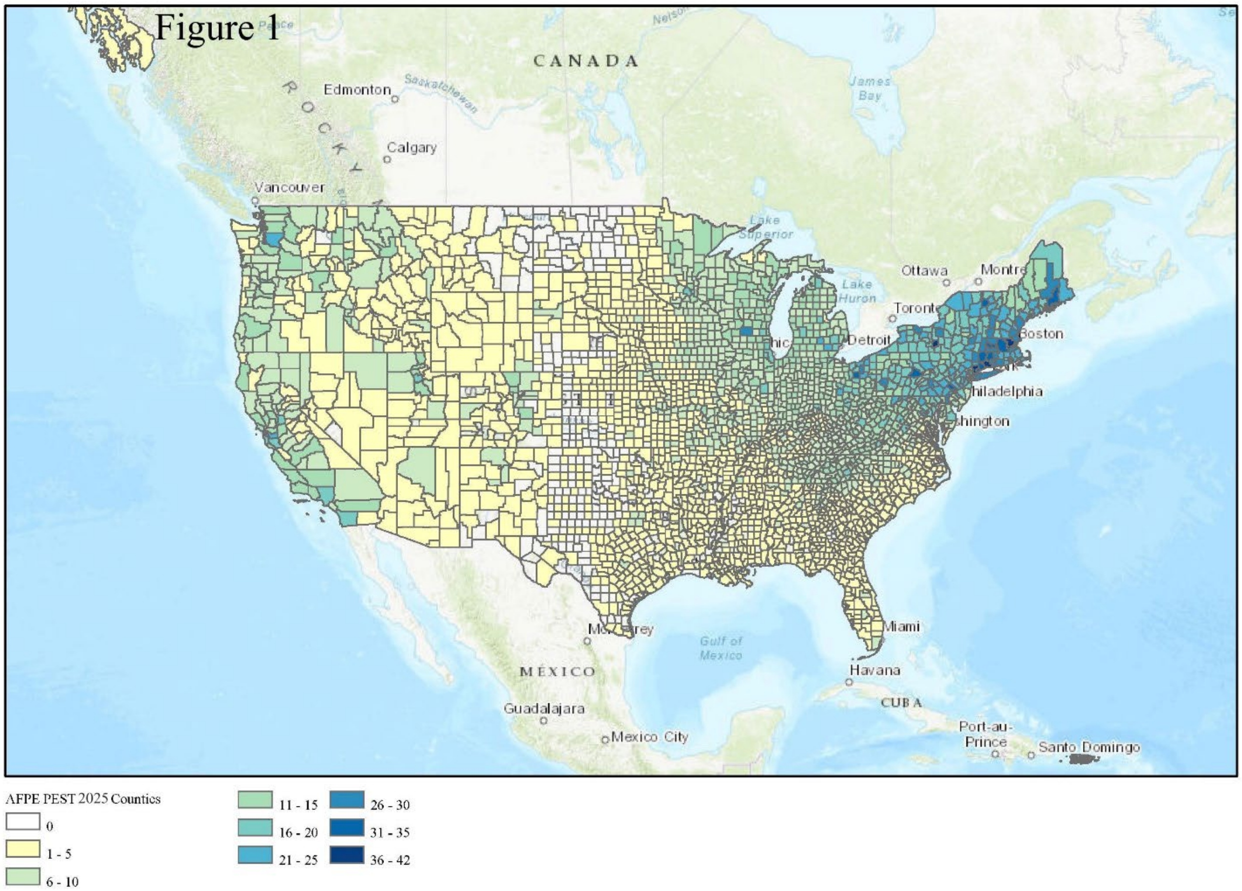

Fig. 1. Map of pest distribution showing total number of pest species detected in each county.

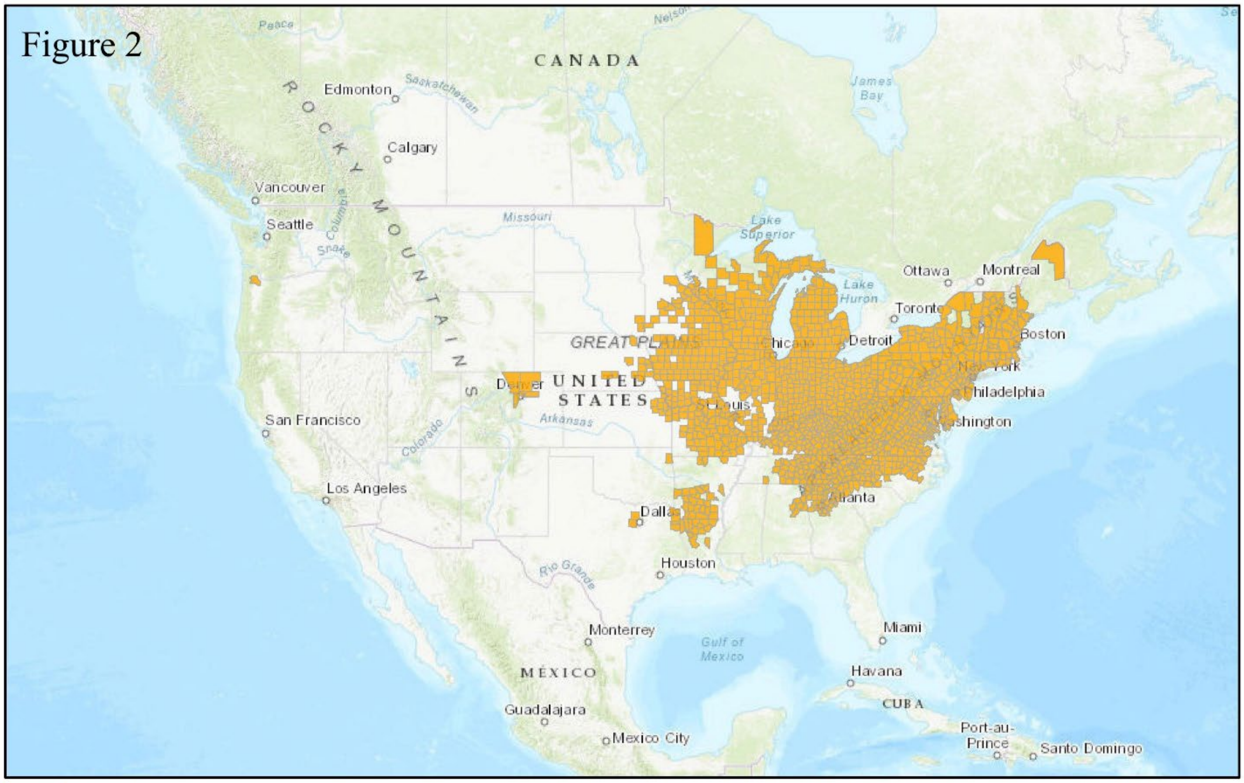

AFPE PEST emerald ash borer 2025 Counties

Fig. 2. Example map of pest distribution for emerald ash borer (*Agrilus planipennis*).

Class I. Data Set Descriptors

A. Data set identity

Alien Forest Pest Detection and Host Volume Statistics for Selected Pests by County in the United States

B. Data set identification code

doi:10.4231/HWQF-V087

C. Data set description

1. Originators

Songlin Fei (Department of Forestry and Natural Resources, Purdue University, West Lafayette, Indiana, USA), Randall S. Morin (USDA Forest Service, Northern Research Station, York, Pennsylvania, USA), Yue S. Li (Libraries and School of Information Studies, Purdue University, West Lafayette, Indiana, USA), Ningning N. Kong (Libraries and School of Information Studies, Purdue University, West Lafayette, Indiana, USA), Susan J. Crocker (USDA Forest Service, Northern Research Station, St. Paul, Minnesota, USA), Frank J. Krist (USDA Forest Service, Forest Health Protection, Fort Collins, Colorado, USA), Katie A. Grong (Department of Forestry and Natural Resources, Purdue University, West Lafayette, Indiana, USA).

2. Abstract

The geographical distributions of established non-indigenous forest insects and pathogens have historically not been well documented nor centrally aggregated, the absence of which has contributed to constrained efforts to manage and minimize resultant damages. To address this need, we present the construction of a centralized database accessible via a publicly available web portal, the Alien Forest Pest Explorer. All non-indigenous forest

insect and pathogen species known to cause economic impacts in the conterminous U.S. were selected for inclusion in the database. The occurrence of these established pests, which is derived from a variety of data streams including reports by state and Federal agencies, the scientific literature, and biodiversity databases, is recorded at the county level. In addition to data on geographical distribution of the pests, the database also provides county-level data that quantifies the volume of host trees species derived from forest inventory data. Overall, the data and analyses presented here provide baseline information regarding the distribution of non-indigenous forest pests and changes in host resources. Our database can also help to provide a more complete understanding of the impacts of non-indigenous forest pests on U.S. forests and can be used as a public resource in supporting regional invasive forest pest research and management. The data are available for reuse under CC0 1.0 Universal licensing; however, we appreciate the citation of this data paper when reusing the dataset.

D. Key words/phrases

Forest insect and disease, Pathway, Pest invasion, Spread, Damage, Mortality

Class II. Research origin descriptors

A. Overall project description

The Alien Forest Pest Explorer (AFPE) is an interactive web tool which provides detailed spatial data describing pest distributions and host inventory estimates for damaging, non-indigenous forest insect and disease pathogens currently established in the United States. To date, the AFPE database includes 76 species of forest insects and 17 species of forest pathogens. This tool allows users to scale county-level data to meet local forest health research needs. The interactive mapping tool contains collective distribution information

for 93 pests, in addition to host specific estimates derived from Forest Inventory and Analysis (FIA) data, including volume, and rates of growth, removals and mortality, for 15 major pest species. A single dataset containing details on harmful, alien forest insects and disease pathogens enables the possibility of prompt and efficient management at a regional level. It aims to serve as a resource for broader, regional strategies to manage invasive forest pests.

1. Identity

   Distributions of non-indigenous forest pests and their impacts on forests in the conterminous United States

2. Originators

   Songlin Fei (Department of Forestry and Natural Resources, Purdue University, West Lafayette, Indiana, USA), Randall S. Morin (USDA Forest Service, Northern Research Station, York, Pennsylvania, USA), Yue S. Li (Libraries and School of Information Studies, Purdue University, West Lafayette, Indiana, USA), Ningning N. Kong (Libraries and School of Information Studies, Purdue University, West Lafayette, Indiana, USA), Susan J. Crocker (USDA Forest Service, Northern Research Station, St. Paul, Minnesota, USA), Frank J. Krist (USDA Forest Service, Forest Health Protection, Fort Collins, Colorado, USA), Katie A. Grong (Department of Forestry and Natural Resources, Purdue University, West Lafayette, Indiana, USA).

3. Period of study

   The dashboard became publicly available in July 2022 and pest distribution data will continue to be updated as data becomes available. Detection year for each

pest is outside the scope of this project, as this information is only available for a small number of species, and insufficient data exists to provide consistent or comprehensive temporal coverage across the full dataset

4. Objectives

The objectives of the dataset include: 1) development of a single dataset describing county-level distribution (i.e. presence/absence) information for 93 known damaging, non-indigenous forest insects and disease pathogens established in the conterminous U.S., and 2) construction of a companion dataset that utilizes forest inventory data from the U.S. Department of Agriculture (USDA), Forest Service, Forest Inventory and Analysis (FIA) program to define resource estimates across the range of hosts for the 15 most damaging forest insect and diseases. The combination of the two datasets allows for analysis of risk based on available host volume and impacts based on changes in host volume across U.S. counties relative to pest and pathogen geographic ranges.

Abstract

The geographical distributions of established non-indigenous forest insects and pathogens have historically not been well documented nor centrally aggregated, the absence of which has contributed to constrained efforts to manage and minimize resultant damages. To address this need, we present the construction of a centralized database accessible via a publicly available web portal, the Alien Forest Pest Explorer. All non-indigenous forest insect and pathogen species known to cause economic and ecological impacts in the conterminous U.S. were selected for inclusion in the database. The occurrence of these established pests,

which is derived from a variety of data streams including reports by state and Federal agencies, the scientific literature, and biodiversity databases, is recorded at the county level. In addition to data on geographical distribution of the pests, the database also provides county-level data that quantifies the volume of host trees species derived from forest inventory data. Overall, the data and analyses presented here provide baseline information regarding the distribution of non-indigenous forest pests, pathogens, and changes in host resources. Our database can also be a resource to help provide a more complete understanding of the impacts of non-indigenous forest pests on U.S. forests and can be used as a public resource in supporting regional invasive forest pest research and management. The data are available for reuse under CC0 1.0 Universal licensing; however, we appreciate the citation of this data paper when reusing the dataset.

5. Sources of funding

This research is partially supported by the US Department of Agriculture Forest Service grant 23-JV-11242305-051

B. Specific subproject description

1. Site description

County level distributions were compiled for all non-indigenous forest insect and pathogen species known to cause economic and ecological impacts in the U.S. This list was updated from the list published by Aukema et al. (2010) which included 62 insect species and 16 forest diseases. The updated list contains 76 insect species and 17 diseases. We applied the same inclusion criteria as used by Aukema et al. (2010), namely the existence of some public document (e.g.,

scientific paper, web page, etc.) documenting damage caused by the species. For insects, the list includes herbivorous species that feed on foliage, sap, wood, or phloem. Species such as predators, pollinators and ants were excluded.

2. Experimental or sampling design

   For major pest species, Forest Health Assessment and Applied Sciences Team (FHAAST) Forest Health Protection produces an annual publication called "Major Forest Insect and Disease Conditions in the U.S.". This national forest pest conditions report highlights the impact of major insects and diseases that cause mortality, defoliation, and other damage to the Nation's forests. Included with those highlights are maps that graphically illustrate the geographic extent of each pest. These maps are compiled at a county level from two main data sources: Aerial Detection Surveys (ADS) and the Pest Event Reporting (PER) database. The PER database is designed to record and track forest pest events at a county level from both unstructured (local knowledge) and structured (formal aerial and ground surveys) pest observations. Narratives that describe each forest pest event are also housed in the PER database. Narratives for pest events deemed nationally significant, are used to develop the national forest pest conditions report. Aerial surveys are conducted using the Digital Mobile Sketch Map system (DMSM). DMSM is tablet hardware, software, and data support processes (ArcGIS Desktop Tools) that allow trained aerial surveyors, in light aircraft, and ground observers to record forest disturbances and their causal agents. Data collected using DMSM aids in the determination of the county level extent for each pest event described in the PER database. Sources such as PER and DMSM generally only record the

presence of damaging populations of a given pest. Such damage is typically limited to a subset of a pest species entire range so these sources tend to only record a portion of a species invaded range. For other pest species, the annual updates have been conducted manually through aforementioned sources such as CAPS Pest Tracker and scholarly publications. New county level presence data were added to the database once it was confirmed. Data for a number of pest species are incomplete due to a lack of reports. Species that cause minor damages and portions of ranges where pest populations are low tend to be under-reported. As a result, there are likely to be significant numbers of false negatives (false absence) recorded in the database. For the more strongly damaging species, this problem is less acute.

3. Research methods

Host tree estimates for the conterminous U.S. are derived from publicly available forest inventory data collected by the USDA Forest Service, FIA program. The FIA sampling design is based on a tessellation of the U.S. into hexagons of ~2,428 ha with at least 1 permanent plot established in each hexagon. Each plot is remeasured every 5 to 10 years, roughly 10% to 20% in each state annually, which provides a statistically robust sampling program for directly estimating tree growth, removal, and mortality rates and appropriate spatial scales. Estimates of host trees are based on inventory data from 92,978 field plots monitored across the US between 2015 and previous years. A copy of code for this extraction can be found in Fei et al 2019. The inventory methodology is described in detail in FIA program documentation (Westfall et al. 2022; Bechtold and Patterson 2005).

4. Project personnel

   Songlin Fei (Department of Forestry and Natural Resources, Purdue University, West Lafayette, Indiana, USA), Randall S. Morin (USDA Forest Service, Northern Research Station, York, Pennsylvania, USA), Yue S. Li (Libraries and School of Information Studies, Purdue University, West Lafayette, Indiana, USA), Ningning N. Kong (Libraries and School of Information Studies, Purdue University, West Lafayette, Indiana, USA), Susan J. Crocker (USDA Forest Service, Northern Research Station, St. Paul, Minnesota, USA), Frank J. Krist (USDA Forest Service, Forest Health Protection, Fort Collins, Colorado, USA), Katie A. Grong (Department of Forestry and Natural Resources, Purdue University, West Lafayette, Indiana, USA).

Class III. Data set status and accessibility

A. Status

1. Latest update

   9/30/2025

2. Latest archive date

   9/30/2025

3. Metadata status

   10/28/2025

4. Data verification

   All data, including user submitted corrections and additions, are checked by experts for validity.

B. Accessibility

1. Storage location and medium

   In addition to this data paper published in *Ecology*, the data are available in the Purdue University Research Repository at https://purr.purdue.edu/publications/4479/1.

2. Contact persons

   Songlin Fei, Department of Forestry and Natural Resources 715 W. State Street, West Lafayette IN, 47907--2061, (765) 496-2199, sfei@purdue.edu

3. Copyright restrictions

4. The data are available for reuse under CC0 1.0 Universal licensing; however, we appreciate the citation of this data paper when reusing the dataset.

5. Proprietary restrictions

   Citation: Researchers should cite this work as follows:

   Fei, S.; Morin, R.; Li, Y.; Kong, N. N.; Crocker, S.; Krist, F.; Liebhold, A.; Grong, K. A. (2024). Alien Forest Pest Detection by Counties in the United States. Purdue University Research Repository. doi:10.4231/HWQF-V087

   Grong, K. A., R. S. Morin, Y. S. Li, N. N. Kong, S. J. Crocker, F. J. Krist, A. M. Liebhold, and S. Fei. 2026. Distributions of economically important non-indigenous forest pests in the conterminous United States. Ecology.

Class IV. Data structural descriptors

A. Data set file

   1. Identity

      FIPS code

   2. Size

15,596 rows, 1.786 MB

3. Format and storage mode

   CSV

4. Header information

   See IV.B. for additional information.

5. Alphanumeric attributes

   Mixed

6. Authentication procedures

   Data is available to be downloaded from a central repository along with supporting documents assuring standardization of information.

B. Variable information

1. Variable identity

   See item B.2 below

2. Variable definition

   Pest distribution tables include the presence of each of all 93 pest species and pathogens in each county. The fields beginning with "DCA" (Damage Causing Agent) and are followed by a five-digit DCA number, which is a unique identifier for the pest species established by the USDA Forest Service (https://www.fs.usda.gov/foresthealth/applied-sciences/mapping-reporting/digital-mobile-sketch-mapping.shtml). It contains presence information, where a value of 1 indicates the presence of the respective species in that county, and a value of 0 indicates its absence. Furthermore, the pest distribution table also has the total number of species and the name of the species for each county. The total number

of species was determined by aggregating the number of species present in each county. The names of the species were compiled by identifying the species' names using DCA. Table 1 provides a description of certain data fields and Table 2 describes the pest definition of each DCA number.

Table 1 Select field names and descriptions

Field name	Description
STATE	State FIPS code (2 digits)
COUNTY	County FIPS code within the state or possession (3 digits)
NAME	County name
LSAD	Legal/statistical area description (LSAD) code
LSAD_TRANS	LSAD description
FIPS	5-digit county FIPS code
Total	Total number of pest species detected
AllPest	Names of pest species detected
DCA[xxxxx]	Presence/absence of the species with the DCA number [xxxxx]. Value 1 for presence, 0 for absence

Table 2 DCA Pest descriptions

DCA Number	Pest	Scientific Name
DCA12208	Ambermarked Birch Leafminer	Profenusa thomsoni
DCA13031	Asiatic Oak Weevil	Cyrtepistomus castaneus
DCA14003	Balsam Woolly Adelgid	Adelges piceae
DCA11057	Banded Elm Bark Beetle	Scolytus schevyrewi Semenov
DCA22042	Beech Bark Disease Complex	Neonectria coccinea var. faginata & Neonectria galligena
DCA14072	Beech Woolly Aphid	Phyllaphis fagi

DCA12048	Birch Casebearer	Coleophora serratella
DCA12070	Birch Leafminer	Fenusa pumila
DCA13016	Black Vine Weevil	Otiorhynchus sulcatus
DCA14086	Bluegum Psyllid	Ctenarytaina eucalypti
DCA12068	Browntail Moth	Euproctis chrysorrhoea
DCA22053	Butternut Canker	Ophiognomonia clavigignenti-juglandacearum
DCA14076	Calico Scale	Eulecanium cerasorum
DCA14077	California Red Scale	Aonidiella aurantii
DCA14089	Camphor Scale	Pseudaonidia duplex
DCA15098	Cherry Bark Tortrix	Enarmonia fidelisana
DCA22023	Chestnut Blight	Cryphonectria parasitica
DCA17023	Chestnut Gall Wasp	Dryocosmus kuriphilus
DCA14078	Circular Hemlock Scale	Lopholeucaspis cinerascens
DCA14079	Cottony Cushion Scale	Icerya purchasi
DCA25020	Dogwood Anthracnose	Elsinoë cornicolans
DCA22087	Dothichiza Canker of Poplar	Dothichiza populea
DCA24022	Dutch Elm Disease	Ophiostoma ulmi
DCA17002	Eastern Spruce Gall Adelgid	Adelges abietis
DCA12210	Elm Flea Weevil	Orchestes steppensis
DCA12141	Elm Leaf Beetle	Xanthogaleruca luteola
DCA12071	Elm Leafminer	Fenusa ulmi Sundeval
DCA14020	Elongate Hemlock Scale	Fiorinia externa

DCA15087	Emerald Ash Borer	Agrilus planipennis
DCA15099	Eucalyptus Longhorned Beetle	Phoracantha semipunctata
DCA15046	Eucalyptus Longhorned Borer	Phoracantha semipunctata
DCA14080	Eucalyptus Psyllid	Glycaspis brimblecombei
DCA12211	Eucalyptus Snout Beetle	Gonipterus scutellatus
DCA25078	Eurasian Poplar Leaf Rust	Melampsora epitea
DCA11061	European Elm Bark Beetle	Scolytus multistriatus
DCA22041	European Larch Canker	Neonectria bruchiana
DCA12212	European Pine Needle Midge	Thecodiplosis piniradiatae
DCA12113	European Pine Sawfly	Neodiprion sertifer
DCA12213	European Spruce Needleminer Moth	Taniva albolineana
DCA12219	European Spruce Sawfly	Gilpinia hercyniae
DCA14081	Golden Oak Scale	Asterolecanium variolosum
DCA14017	Green Spruce Aphid	Cinara fornacula
DCA14004	Hemlock Woolly Adelgid	Adelges tsugae
DCA12215	Imported Willow Leaf Beetle	Plagiodera versicolora
DCA12153	Introduced Basswood Thrips	Thrips calcaratus
DCA12061	Introduced Pine Sawfly	Diprion similis
DCA12138	Japanese Beetle	Popillia japonica
DCA15100	Japanese Cedar Longhorn Beetle	Callidiellum rubicundum
DCA14082	Juniper Scale	Carulaspis juniperi
DCA12047	Larch Casebearer	Coleophora laricella

DCA12139	Larch Sawfly	Pristiphora erichsonii
DCA24031	Laurel Wilt	Raffaelea lauricola
DCA14087	Linden Aphid	Eucallipterus tiliae
DCA21019	Littleleaf Disease	Phytophthora cinnamomi
DCA12034	Maple Petiole Borer	Caulocampus acericaulis
DCA11060	Mediterranean Pine Engraver	Orthotomicus erosus
DCA12216	Mimosa Webworm	Homadaula anisocentra
DCA12140	Mountain-Ash Sawfly	Pristiphora geniculata
DCA14088	Norway Maple Aphid	Periphyllus lyropictus
DCA14085	Oak Pit Scale	Asterodiaspis minor
DCA24021	Oak Wilt	Bretziella fagacearum
DCA14028	Oystershell Scale	Lepidosaphes ulmi
DCA15101	Peach Twig Borer	Anarsia lineatella
DCA12030	Pear Sawfly	Caliroa cerasi
DCA14058	Pear Thrips	Taeniothrips inconsequens
DCA12010	Pine False Webworm	Acantholyda erythrocephala
DCA28001	Pine Shoot Beetle	Eucosma gloriola
DCA14083	Pink Hibiscus Mealybug	Maconellicoccus hirsutus
DCA12217	Poplar sawfly	Cladius grandis
DCA15019	Poplar-And-Willow Borer	Cryptorhynchus lapathi
DCA21020	Port-Orford-Cedar Root Disease	Phytophthora lateralis
DCA12218	Red Gum Lerp Psyllid	Glycaspis brimblecombei
DCA14033	Red Pine Scale	Matsucoccus matsumurae

DCA11062	Redhaired Pine Bark Beetle	Hylurgus ligniperda
DCA14084	San Jose Scale	Comstockaspis perniciosus
DCA12086	Satin Moth	Leucoma salicis
DCA22034	Scleroderris Canker	Gremmeniella abietina
DCA15090	Sirex Woodwasp	Sirex noctilio
DCA11045	Smaller European Elm Bark Beetle	Scolytus multistriatus
DCA15102	Soapberry Borer	Agrilus biguttatus
DCA12089	Spongy Moth	Lymantria dispar
DCA14044	Spruce Bud Scale	Physokermes piceae
DCA13015	Strawberry Root Weevil	Otiorhynchus ovatus
DCA26001	White Pine Blister Rust	Otiorhynchus ovatus
DCA22088	Willow Scab	Venturia saliciperda
DCA12197	Winter Moth	Operophtera brumata
DCA25092	Beech Leaf Disease	Litylenchus crenatae
DCA14090	Spotted Lanternfly	Lycorma delicatula
DCA90001	Elm Zig-zag Sawfly	Aproceros leucopoda
DCA90002	Crepe Myrtle Scale	Lepidosaphes cryptera
DCA90003	Polyphagous Shothole Borer	Euwallacea fornicatus
DCA18030	European pine shoot moth	Thecodiplosis piniradiatae
DCA22091	Sudden Oak Death	Phytophthora ramorum

3. Data type

   a. Storage type

      Integer

   b. List and definition of variable codes

      0 means absence, 1 means presence

4. Data format

   a. Columns

      Start column “OBJECTID”, end column “SPECIES_SCIENTIFIC_NAME”

C. Data anomalies

Data is updated annually and the most recent data from each U.S. State is included. County-level estimates of host volume, growth, mortality, and removals including sampling errors are included.

Class V. Supplemental descriptors

A. Data acquisition

1. Data forms or acquisition methods

   Forest inventory data are generated from the public database of the Forest Service’s Forest Inventory and Analysis (FIA) Program (FIADB). The USDA Animal and Plant Health Inspection Service (APHIS) publishes annual county-level data on the known distribution for certain high-profile species. The USDA Forest Service annually publishes a national ‘Pest Conditions Report’ that provides distributional data for several non-indigenous forest pest species. From these various sources of pest distribution data, we recorded the current presence–

absence of each pest species for individual U.S. counties. For other pest species, it was necessary to assemble their ranges from a mixture of sources. These included the Cooperative Agricultural Pest Survey (CAPS) Pest Tracker (https://pest.ceris.purdue.edu/pests.php), the Bark and Ambrosia Beetles of the Americas webpage (Atkinson 2022), the Global Biodiversity Information Facility (GBIF 2022), iNaturalist research grade observations (iNaturalist 2022), BugGuide (VanDyk 2020) and other scientific papers found using Google Scholar searches.

2. Data entry verification procedures

   See Class V.B below.

B. Quality assurance/quality control procedures

   The Alien Forest Pest Explorer (AFPE) web site (https://mapsweb.lib.purdue.edu/AFPE/) contains a "contact us" link where users are able to send in corrections and additions that are checked by experts for validity.

C. Related materials

   US county shapefile

D. Computer programs and data-processing algorithms

   ArcGIS to join data to US county shapefile.

E. Archiving

   1. Archival procedures

      Data are archived by Purdue University Research Repository (PURR)

F. History of data set usage

   1. Data request history

The data has 865 total views and 223 total downloads (10/28/2025).

2. Data set update history

   Data was last updated 9/2025

Literature Citations

Atkinson, T. H. n.d. “Bark and Ambrosia Beetles of the Americas.” Last accessed October, 2024. http://www.barkbeetles.info.

Aukema, Juliann E., Deborah G. McCullough, Betsy Von Holle, Andrew M. Liebhold, Kerry Britton, and Susan J. Frankel. 2010. “Historical Accumulation of Nonindigenous Forest Pests in the Continental United States.” *Bioscience* 60 (11): 886–97.

Bechtold, W.A.; Patterson, P.L., eds. 2005. The enhanced Forest Inventory and Analysis Program—national sampling design and estimation procedures. Gen. Tech. Rep. SRS-80. Asheville, NC: U.S. Department of Agriculture, Forest Service, Southern Research Station. 85 p. https://doi. org/10.2737/SRS-GTR-80.

Brockerhoff, E. G., & Liebhold, A. M. (2017). Ecology of forest insect invasions. *Biological Invasions*, *19*, 3141-3159.

“BugGuide” BugGuide. Last accessed October, 2024. https://bugguide.net/user/view/612

Fei, Songlin, Randall S. Morin, Christopher M. Oswalt, and Andrew M. Liebhold. 2019. “Biomass Losses Resulting from Insect and Disease Invasions in US Forests.” *Proceedings of the National Academy of Sciences of the United States of America* 116 (35): 17371–76.

“GBIF.” GBIF. Last accessed October, 2024. https://www.gbif.org/

Guo, Qinfeng, Songlin Fei, Kevin M. Potter, Andrew M. Liebhold, and Jun Wen. "Tree diversity regulates forest pest invasion." *Proceedings of the National Academy of Sciences* 116, no. 15 (2019): 7382-7386.

Hudgins, Emma J., Andrew M. Liebhold, and Brian Leung. "Predicting the spread of all invasive forest pests in the United States." *Ecology letters* 20, no. 4 (2017): 426-435.

“INaturalist.” iNaturalist. Last accessed October, 2024. https://www.inaturalist.org.

Liebhold, Andrew M., Deborah G. McCullough, Laura M. Blackburn, Susan J. Frankel, Betsy Von Holle, and Juliann E. Aukema. 2013. “A Highly Aggregated Geographical Distribution of Forest Pest Invasions in the USA.” *Diversity & Distributions* 19 (9): 1208–16.

Liebhold, Andrew M., Eckehard G. Brockerhoff, Susan Kalisz, Martin A. Nuñez, David A. Wardle, and Michael J. Wingfield. "Biological invasions in forest ecosystems." *Biological Invasions* 19, no. 11 (2017): 3437-3458.

Lovett, Gary M., Marissa Weiss, Andrew M. Liebhold, Thomas P. Holmes, Brian Leung, Kathy Fallon Lambert, David A. Orwig et al. "Nonnative forest insects and pathogens in the United States: Impacts and policy options." *Ecological applications* 26, no. 5 (2016): 1437-1455.

Morin, R.S.; Liebhold, A.M. 2016. Invasive forest defoliator contributes to the impending downward regional trend of oak dominance in eastern North America. Forestry: An International Journal of Forest Research. 89: 284-289.

Siegert, N.W.; McCullough, D.G.; Luther, T.; Benedict, L.; Crocker, S.; Church, K.; Banks, J. 2023. Biological invasion threatens keystone species indelibly entwined with Indigenous cultures. Frontiers in Ecology and the Environment. 21(7): 310-316.

Trotter, R.T.; Morin, R.S.; Liebhold, A.M.; Oswalt, S.N. 2013. Changes in the regional abundance of hemlock associated with hemlock woolly adelgid (Adelges tsugae Annand) invasion. Biological Invasions 15: 2667-2679.

Ward, Samuel F., Andrew M. Liebhold, and Songlin Fei. "Variable effects of forest diversity on invasions by non-native insects and pathogens." *Biodiversity and Conservation* 31, no. 11 (2022): 2575-2586.

Westfall, James A., John W. Coulston, Gretchen G. Moisen, and Hans-Erik Andersen. 2022. “Sampling and Estimation Documentation for the Enhanced Forest Inventory and Analysis Program: 2022.” Edited by James A. Westfall, John W. Coulston, Gretchen G. Moisen, and Hans-Erik Andersen. Madison, WI: U.S. Department of Agriculture, FoFerest Service, Northern Research Station. https://doi.org/10.2737/nrs-gtr-207.
